# A Three-Dimensional Microdisplacement Sensing System Based on MEMS Bulk-Silicon Technology

**DOI:** 10.3390/s141120533

**Published:** 2014-10-30

**Authors:** Junjie Wu, Lihua Lei, Xin Chen, Xiaoyu Cai, Yuan Li, Tao Han

**Affiliations:** 1 School of Electronic Information and Electrical Engineering, Shanghai Jiao Tong University, No. 800, Dongchuan Road, Shanghai 200240, China; E-Mails: wujunjiesimt@gmail.com (J.W.); than@sjtu.edu.cn (T.H.); 2 Shanghai Institute of Measurement and Testing Technology, National Center of Measurement and Testing for East China, National Center of Testing Technology, No. 1500, Zhangheng Road, Shanghai 201203, China; E-Mails: leilh@simt.com.cn (L.L.); caixiaoyu@simt.com.cn (X.C.); liyuan@simt.com.cn (Y.L.)

**Keywords:** dimensional metrology, piezoresistor, MEMS, microtactile sensor

## Abstract

For the dimensional measurement and characterization of microsized and nanosized components, a three-dimensional microdisplacement sensing system was developed using the piezoresistive effect in silicon. The sensor was fabricated using microelectromechanical system bulk-silicon technology, and it was validated using the finite element method. A precise data acquisition circuit with an accuracy of 20 μV was designed to obtain weak voltage signals. By calibration, the sensing system was shown to have a sensitivity of 17.29 mV/μm and 4.59 mV/μm in the axial and lateral directions, respectively; the nonlinearity in these directions was 0.8% and 1.0% full scale, respectively. A full range of 4.6 μm was achieved in the axial direction. Results of a resolution test indicated that the sensing system had a resolution of 5 nm in the axial direction and 10 nm in the lateral direction.

## 1. Introduction

Miniaturized components offer advantages such as a smaller volume, lighter weight, lower energy consumption, and more functionality; moreover, they are generally more robust for both consumer and industrial applications. Dimensional metrology for micro- and nanosized components is becoming increasingly important in industries such as the semiconductor and optical industries, as well as in the fields of mechanical engineering, biology, and medicine. The dimensional measurement of microsystem components is a field of application whose importance is increasing [1–3].

A coordinate measuring machine (CMM) is a good tool for the three-dimensional (3D) characterization of mechanical parts. However, the traditional CMM has limited resolution and results in uncertainties when applied to micro- and nanoscale measurements. Recently, ultraprecision CMM with a tactile probe have been developed. Several three-dimensional microtactile probes have been developed based on different principles [4–8]. In this paper, a 3D microdisplacement sensing system constructed using MEMS technology is presented. A data acquisition circuit was designed for the sensor. Performance tests for the range, linearity, hysteresis, and resolution were conducted.

Compared to an identical piezoresistive-principle-based design presented in [7], the sensor developed in the present study here had less piezoresistors, which simplified the detection circuit in a limited area of sensitive beams. Moreover, the cross-beam structure had a lower contact force, and the stress caused by deflection was more concentrated in the beams, which led to a higher output voltage and therefore to a higher sensitivity. Similar cross-beams can be seen in the force sensor presented by Vasarhelyi [9–11] and the force-moment sensor developed by Dao [12]. These two sensors were specially designed to detect force.

## 2. Theory and Modelling

**Figure 1.**
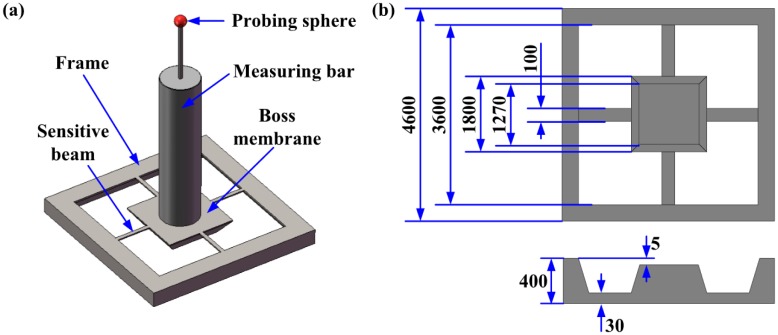
Mechanical structure. (**a**) Mechanical structure; (**b**) Detailed size /μm.

Since the discovery of the piezoresistive effect in silicon by Smith in 1954, it has been widely used in many types of sensors [13]. The 3D microdisplacement sensing system presented in this paper was also based on this phenomenon. The sensing system consisted of a cross-shaped sensitive beam, a 6.8 mm long stylus with a probing sphere of 300 μm diameter, and three Wheatstone bridges, which were connected to a precise data acquisition circuit. The mechanical structure is shown in Figure 1.

### 2.1. Displacement Detection

**Figure 2.**
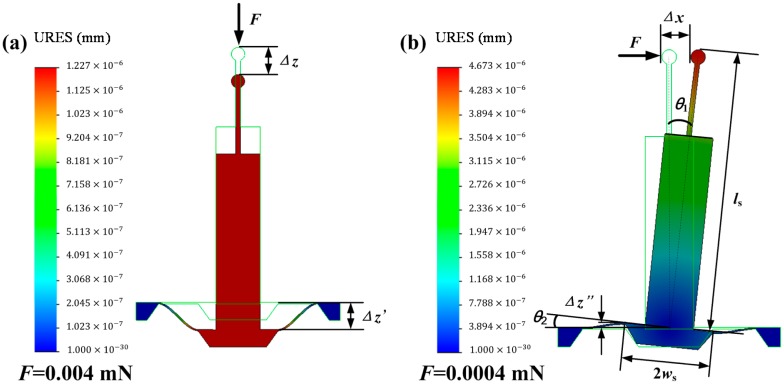
Displacement detection model. (**a**) Axial displacement; (**b**) Lateral displacement.

To illustrate the measurement mechanism, a displacement detection model built using SolidWorks is shown in Figure 2. When loaded with an axial or lateral load, the shifting of the probing sphere leads to the shifting of the boss membrane through the measuring bar, which in turn causes the shifting or rotation of the boss membrane. This deforms the sensitive beams and the stresses in the beams are changed. Variations in the stresses can be obtained by measuring the resistance changes in the resistors in the sensitive beams.

The material of the sphere was ruby, and the measuring bar was made of tungsten carbide. The thickness of the boss membrane was 395 μm, which was approximately ten times thicker than the sensitive beam, whose thickness was 30 μm. Thus, the stiffness of the boss membrane was far greater than that of the sensitive beam. This was verified by performing a stress simulation (discussed in Section 2.2). The stylus and boss membrane were connected by epoxy resin. Ruby, tungsten carbide, and epoxy resin have relatively high stiffness values. In other words, the deformation of the displacement transmission system, which consisted of the stylus and boss membrane, could be neglected.

When the sphere is axially displaced by Δ*z*, a shift of Δ*z′* is generated between the two ends of the sensitive beam, as shown in Figure 2a. Considering the rigidity assumptions for the epoxy resin, stylus, and boss membrane, Δ*z′* can be viewed as being equal in value to Δ*z*. Figure 2b shows the lateral displacement detection model. The shift of Δ*z″* between the two ends of the sensitive beam is approximately determined using the expression:

(1) $$\Delta z^{\prime \prime }=w_{\text{s}}\times \frac{\Delta x}{l_{\text{s}}}$$

where *w*_s_ is the half-width of the boss membrane, Δ*x* is the lateral displacement of the sphere, and *l*_s_ is the length of the stylus. When Δ*x* is imparted to the sphere, a rotation (by angle *θ*_1_) is generated, causing a rotation (by angle *θ*_2_) of the boss membrane. Regardless of the deformation in the transmission system, *θ*_2_ can be seen as being equal in value to *θ*_1_.

### 2.2. Stress Simulation

**Figure 3.**
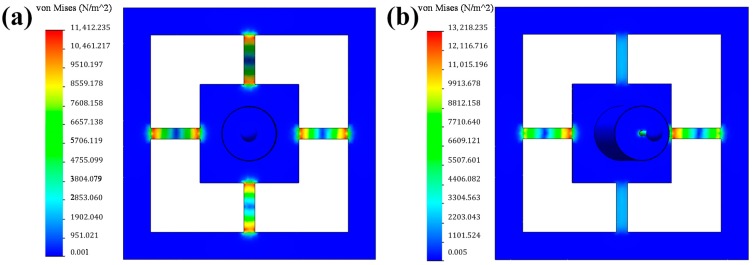
Stress distribution. (**a**) Axial load; (**b**) Lateral load.

To realize optimal performance, stress simulation was conducted using a finite element method (FEM). Forces of 0.004 and 0.0004 mN were applied in the axial and lateral directions, respectively. The results can be observed in Figure 3. The design parameters of the sensing unit were determined using the FEM. The detailed analysis of the stiffness was similar to that performed in [14].

When a load was placed on the sphere, normal stress was mainly concentrated on the sensitive beam. There was nearly zero stress on the frame and boss membrane. Therefore, the resistors should be distributed at the two ends of each sensitive beam, where there is maximum stress.

### 2.3. Acquisition Circuit Design

**Figure 4.**
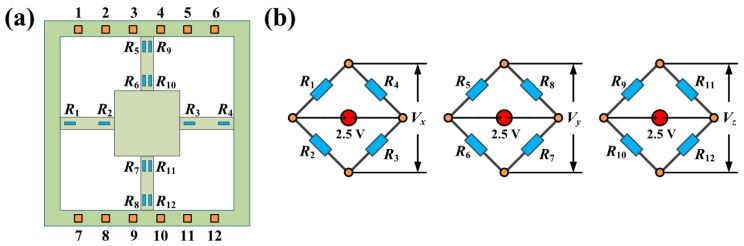
Stress detection circuit. (**a**) Resistors distribution; (**b**) Wheatstone bridge.

Variations in the stresses in the sensitive beam were detected by the three Wheatstone bridges, each of which consisted of four resistors. The resistors were easily affected by thermal drift. Therefore, the Wheatstone bridge was effective in improving the sensitivity. Figure 4 shows the distribution of the resistors and the connections of each resistor. The outputs of the axial and lateral loads were detected by different bridges. *R*_1_–*R*_4_ were part of the first Wheatstone bridge, which was used to detect the *X*-direction stress. *R*_5_–*R*_8_ were used for *Y*-direction stress detection, and *R*_9_–*R*_12_ were used for *Z*-direction stress detection.

**Figure 5.**
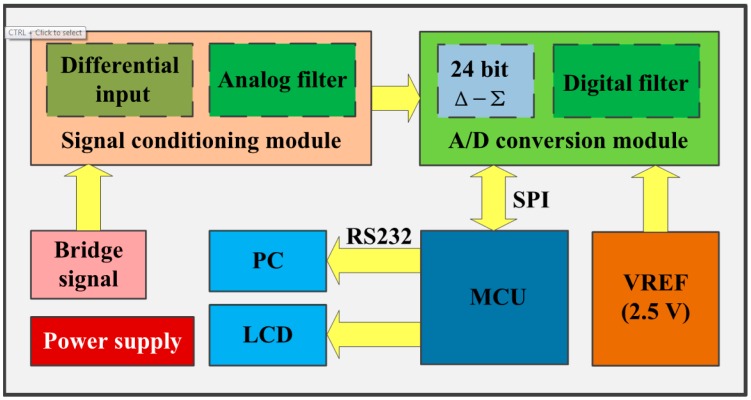
Structure of data acquisition system.

A precise data acquisition system was necessary to achieve a weak voltage signal output in the Wheatstone bridge. The system frame is shown in Figure 5. A precision linear voltage regulator module was designed to ensure stable power supply. The bridge signal was applied difference disposal and analogue filtering before analogue-to-digital conversion. After conversion, digital filtering was performed. ADS1274 was selected as the voltage-to-digital converter, and it has a theoretical resolution of 0.149 μV. The results could be displayed directly on a liquid crystal display or on a software interface by using a personal computer, which made it easy to record and process the results. The full range of the system was ±2.5 V. Owing to the circuit and environmental noise, the acquisition accuracy achieved was 20 μV.

The relative change in the resistance of the resistor as a result of normal stress can be approximately described by the expression:

(2) $$\frac{\Delta R}{R}=\frac{1}{2}(\pi_{11}+\pi_{12}+\pi_{44})\times \sigma_{1}=71.8\times 10^{-11}\times \sigma_{1}$$

where σ_1_ is the normal stress, and π_11_, π_12_, and π_44_ are the piezoresistance coefficients. For a bulk p-type piezoresistor with a resistivity of 7.8 Ω·cm, these coefficients are π_11_ = 6.6 × 10^−11^ Pa^−1^, π_12_ = −1.1 × 10^−11^ Pa^−1^, and π_44_ = 138.1 × 10^−11^ Pa^−1^ [12].

The output voltage of the Wheatstone bridge *V*_out_ is given by:

(3) $$V_{\text{out}}=\frac{\Delta R}{R}\times V_{\text{in}}$$

where *V*_in_ is the input voltage of the Wheatstone bridge. In this study, the input voltage was 2.5 V.

When using an acquisition accuracy of 20 μV as *V*_out_ and an input voltage of 2.5 V as *V*_in_ in Equation (3), a Δ*R*/*R* value of 8 × 10^−6^ is obtained. Subsequently, using this Δ*R*/*R* value in Equation (2), a σ_1_ value of 1.1142 × 10^4^ Pa is obtained. A stress smaller than 1.1142 × 10^4^ Pa was not detected. Therefore, forces of 0.004 and 0.0004 mN were applied in the simulation. The simulation results in Figure 2 show that theoretical resolutions of 1.227 and 4.673 nm were achieved for the axial and lateral directions by using FEM.

## 3. Fabrication

**Figure 6.**
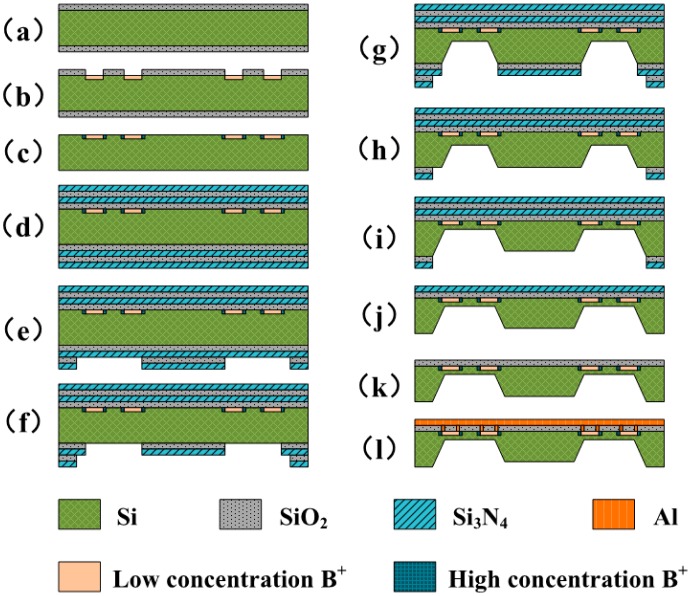
Fabrication process of the sensing unit. (**a**) The starting material was an n-type silicon wafer with a thickness of 400 μm. After cleaning the substrate, a 3000 Å-thick SiO_2_ insulator layer was formed using a thermal oxidization process; (**b**) The shapes of the piezoresistors were patterned using the photoetching process. Photoresist was used as a mask and the SiO_2_ layer was etched using reactive ion etching (RIE). A low-concentration of boron ions was diffused to form p-type piezoresistors; (**c**) A high-concentration of boron ions was diffused at both ends of each piezoresistor. This process was similar to (**b**); (**d**)–(**f**) Two layers of SiO and Si_3_N_4_ were deposited using low-pressure chemical vapour deposition. After performing photoetching and corrosion of Si_3_N_4_ and SiO two times, a corrosion window of KOH was formed and the silicon substrate was exposed; (**g**)–(**i**) The first KOH corrosion was performed at a depth of 365 μm. Subsequently, the outer layers of Si_3_N_4_ and SiO were removed. A 5 μm gap was introduced between the boss membrane and the rear panel after the second KOH corrosion; (**j**)–(**k**) The outer layers of Si_3_N_4_ and SiO were removed on both sides, and the Si_3_N_4_ layer was etched on the upper side; (**l**) Wire holes were patterned and etched using the photoetching and RIE processes. Aluminium wires and bonding pads were formed through vacuum evaporation, photolithography, and etching processes. Finally, the structure was freed using the inductively coupled plasma process.

The sensing unit was fabricated using an MEMS bulk-silicon process, which is shown in Figure 6.

**Figure 7.**
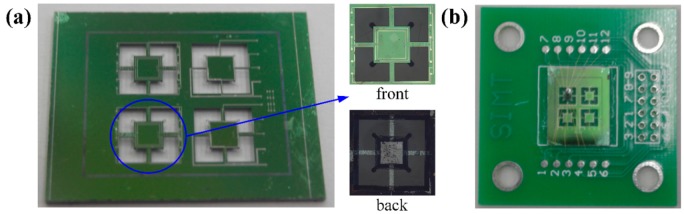
Picture of manufactured sensor. (**a**) Sensing unit; (**b**) Assembled sensor.

Figure 7a shows the manufactured sensing unit, which was fixed on a printed circuit board and connected to the data acquisition circuit through gold wires. The size of each unit was 4.6 mm × 4.6 mm. The assembled sensor is shown in Figure 7b. The value of the fabricated sheet resistor was approximately 5.83 kΩ.

## 4. Calibration and Analysis

**Figure 8.**
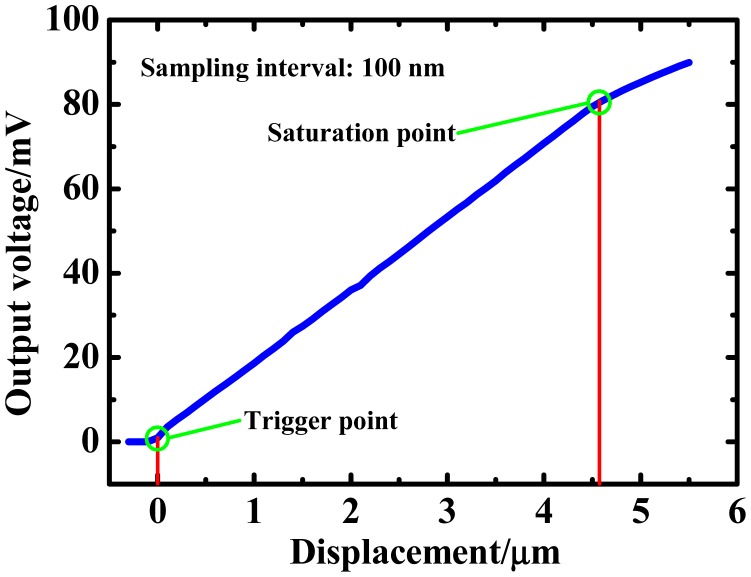
Axial range calibration.

The sensor was calibrated using a nanometre positioning system, which has been described in [15]. The moving stage had a large coarse positioning range of 25 mm × 25 mm × 10 mm, a precise uniaxial positioning range of 12 μm, and an accuracy of 1 nm. A charge-coupled device camera was used as an auxiliary observation tool. The precise uniaxial positioning stage was calibrated by laser interferometer before experiment. The measurement error of the laser interferometer is within 0.7 × 10^−6^ *L*, where *L* is the measurement range. Figure 8 shows the result of axial range calibration. The sensor had an axial range of 4.6 μm. The saturation point indicates that the boss membrane was in touch with the backboard. The lateral range was not tested because of the risk of fracture. However, from (1), it can be deduced that the lateral range was larger than the axial range.

**Figure 9.**
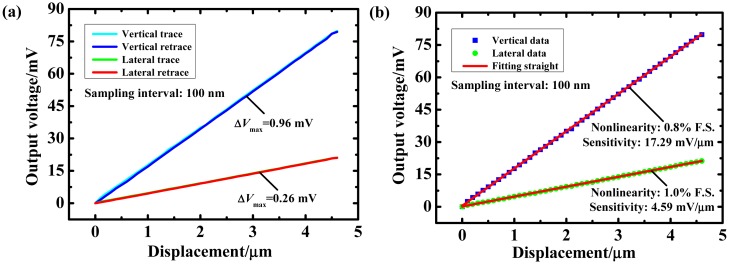
Hysteresis and linearity test. (**a**) Hysteresis test; (**b**) Linearity test.

The hysteresis and linearity were tested to determine the output performance of the sensor (see Figure 9). The axial and lateral hystereses were 0.96 mV and 0.26 mV. Furthermore, the sensor had sensitivity values of 17.29 mV/μm and 4.59 mV/μm during axial and lateral approach, respectively; the nonlinearity values for these approaches were 0.8% full scale (FS) and 1.0% FS, respectively. The linearity was evaluated using linear regression. The R^2^ values were 0.99993 for axial fitting and 0.99981 for lateral fitting.

**Figure 10.**
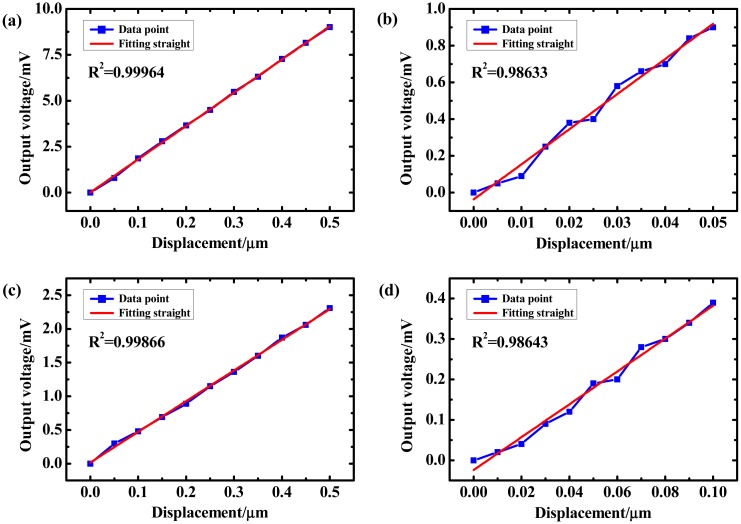
Resolution test. (**a**) Vertical 50 nm step; (**b**) Vertical 5 nm step; (**c**) Lateral 50 nm step; (**d**) Lateral 10 nm step.

Resolution is an important parameter that directly determines the probing precision of a sensor. In the resolution test, the trigger point was set as the starting position. For determining the axial resolution, 50 and 5 nm steps were chosen. Figure 10b shows that the linearity of the straight fitting was 0.98633, which is an acceptable value. For an approaching step less than 5 nm, the linearity of the straight fitting decreased sharply, and data values of the forward and backward points could not be distinguished. Therefore, the axial resolution was regarded as 5 nm. Similarly, the lateral resolution was determined to be 10 nm. Cross-talk was not tested here; it may be mentioned here that cross-talk was observed to be very weak [14].

Additionally, there is another question that should be discussed here. Compared to the triskelion designs by Bos and Claverley, the cross-shape design is over constrained [1,5]. It may have a large effect to the measurement results as the flexure is not full rotational symmetry. However, the decoupling is easier in the cross-shape design. There are two ways to eliminate the influence of non-rotational symmetry. The first way is to calibrate the sensor in x, y and z direction respectively by the nano measuring machine before measurement and apply the obtained input-output curve to calculate the measurement results. The second way is to develop the related algorithm to eliminate the error. The angle of non-rotational symmetry can be calculated by measuring the output voltages of *x*, *y* and *z* axes simultaneously. Then, the calculated angle could be used to correct the measurement results. If the second method is realized, the sensing system can measure displacement in an arbitrary vector in the *x*-*y* plane.

## 5. Conclusions

A 3D microdisplacement sensing system was designed and fabricated using MEMS bulk-silicon technology. A displacement detection model was constructed, and stress distribution was simulated using a FEM. A circuit with three Wheatstone bridges was used to measure the variation in stress and a precise data acquisition system was developed. By calibration, the sensing system was shown to have sensitivity values of 17.29 and 4.59 mV/μm in the axial and lateral directions, respectively; the nonlinearity values in these directions were 0.8% and 1.0% FS, respectively. An axial hysteresis of 0.96 mV and a lateral hysteresis of 0.26 mV were obtained. The results of the resolution test indicated that the sensing system had a 5 nm resolution in the axial direction and a 10 nm resolution in the lateral direction. In future work, aspects such as the probing force, thermal drift, compensations, and repeatability will be investigated. Integration with a nanoscale measuring and positioning platform will be performed by developing an associated hardware interface and software.
